# Supplementary material for: Nitrogen oxides under pressure: stability, ionization, polymerization, and superconductivity
Source: Sci Rep. 2015 Nov 17;5:16311. doi: 10.1038/srep16311 (PMC4648296; doi:10.1038/srep16311)
Supplement: Supplementary Information [file srep16311-s1.doc]

**Supplementary Materials：****Nitrogen oxides under pressure: stability, ionization, polymerization, and superconductivity**

Dongxu Li^1,*^, Artem R. Oganov^2,3,4,5,6^, Xiao Dong^7^, Xiang-Feng Zhou^3,4,7^, Qiang Zhu^3,4^, Guangrui Qian^3,4^, Huafeng Dong^3,4^

*^1^ College of Materials Science and Engineering, Huaqiao University, Xiamen, 361021 P.R. China*

*^2^ Skolkovo Institute of Science and Technology, Skolkovo Innovation Center, 3 Nobel St., Moscow 143026, Russia*

*^3^ Department of Geosciences, Stony Brook University, Stony Brook, NY 11794, USA*

*^4^ Center for Materials by Design, Institute for Advanced Computational Science, Stony Brook University, Stony Brook, NY 11794, USA*

*^5^Moscow Institute of Physics and Technology, 9 Institutskiy lane, Dolgoprudny city, Moscow Region, 141700, Russia*

*^6^ School of Materials Science and Engineering, Northwestern Polytechnical University, Xi’an, 710072, China*

*^7^ School of Physics and Key Laboratory of Weak-Light Nonlinear Photonics, Nankai University, Tianjin 300071, China*

*^*^Corresponding author: lidongxu@hqu.edu.cn*


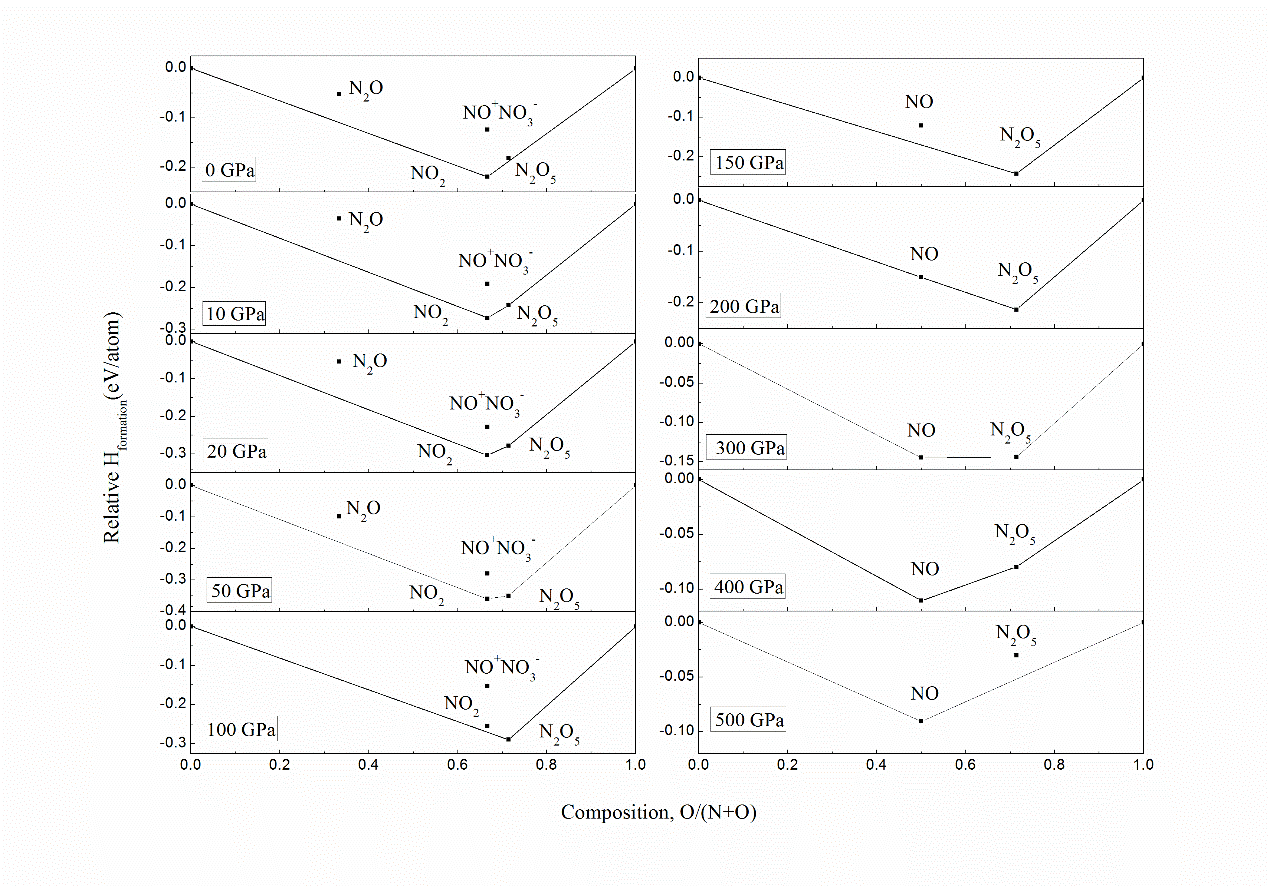


Figure S1 Convex hull diagram of N-O system at given pressure and zero temperature.

Table S1 Novel Structures of NO_2_, N_2_O_5_, NO and NO^+^NO_3_^-^.

| Phases | Pressure  (GPa) | Lattice parameters  (Å) | Atomic coordinates |
| --- | --- | --- | --- |
| *P*2_1_/*c* NO_2_ (No.14) | 65 | *a*=5.823  *b*=5.566  *c*=5.071  *β*=82.8º | N1 0.5790 0.5809 1.0728  N2 0.0990 0.5624 0.5679  O1 0.7779 0.9621 0.5126  O2 0.5303 0.2827 0.7802  O3 0.2821 0.0262 1.0041  O4 0.9686 0.2825 1.2822 |
| *P*-1 N_2_O_5_ (No.2) | 10 | *a*=4.525  *b*=5.435  *c*=6.497  *β*=113.5º | N1 0.0986 0.0367 0.7788  N2 0.3359 0.4515 0.2088  O1 0.4251 0.3923 0.6424  O2 0.9313 0.5578 0.7899  O3 0.5995 0.7415 0.9875  O4 0.7867 0.1039 0.3574  O5 0.1587 0.9437 0.2130 |
| *C*2*/c* N_2_O_5_ (No.15) | 55 | *a*=10.8  *b*=4.076  *c*=4.751  *β*=68.7º | N1 0.8909 0.7828 0.9631  O1 0.8126 0.4007 0.6284  O2 0.8943 0.9267 0.4486  O3 0.5000 0.9050 0.2500 |
| *P*2_1_*/m* NO (No.11) | 200 | *a*=3.100  *b*=2.148  *c*=2.974  *β*=75.0º | N1 0.0301 0.2500 0.1213  O1 0.3331 0.2500 0.3018 |
| *P*2_1_ NONO_3_ (No.4) | 20 | *a*=4.708  *b*=4.975  *c*=4.592  *β*=90.7º | N1 0.4558 0.3994 0.6447  N2 0.9974 0.7389 0.7549  O1 0.3976 0.2377 0.8031  O2 0.8487 0.9476 0.7552  O3 0.8929 0.5059 0.7442  O4 0.2684 0.7623 0.7707 |


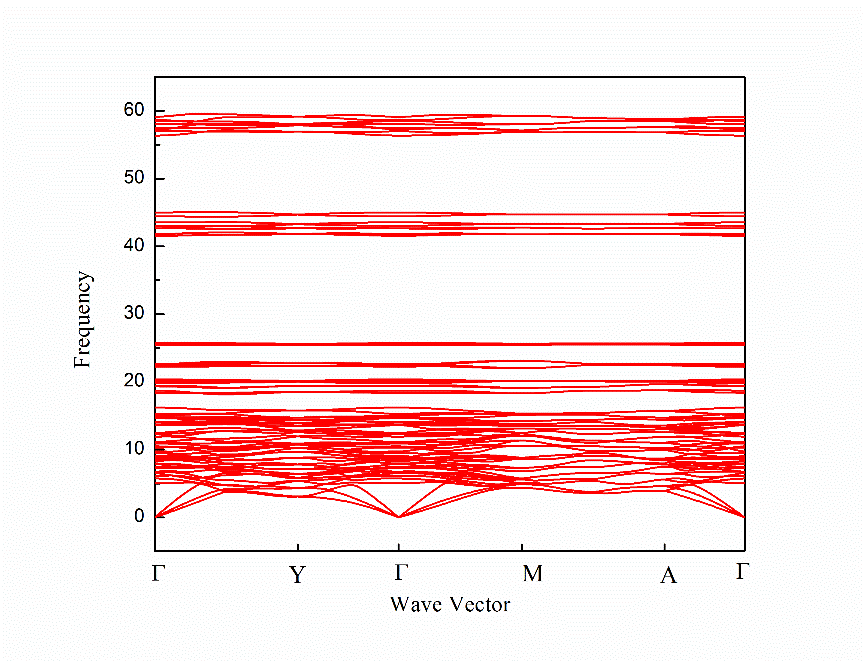


Figure S2 Phonon dispersion curves of *P*2_1_*/c* NO_2_. Γ(0,0,0) Y(0.5,0.5,0) M(0.5 0.5 0.5) A(0,0.5,0.5)


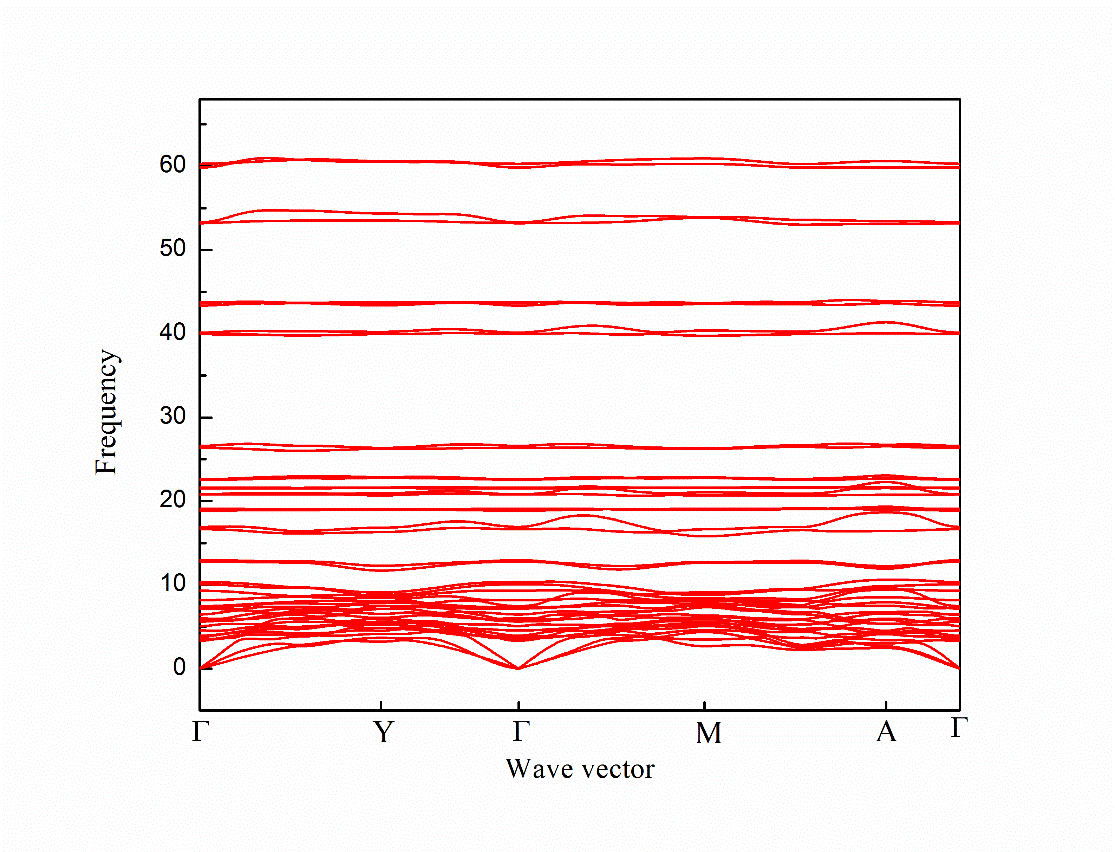


Figure S3 Phonon dispersion curves of *P*-1 N_2_O_5_
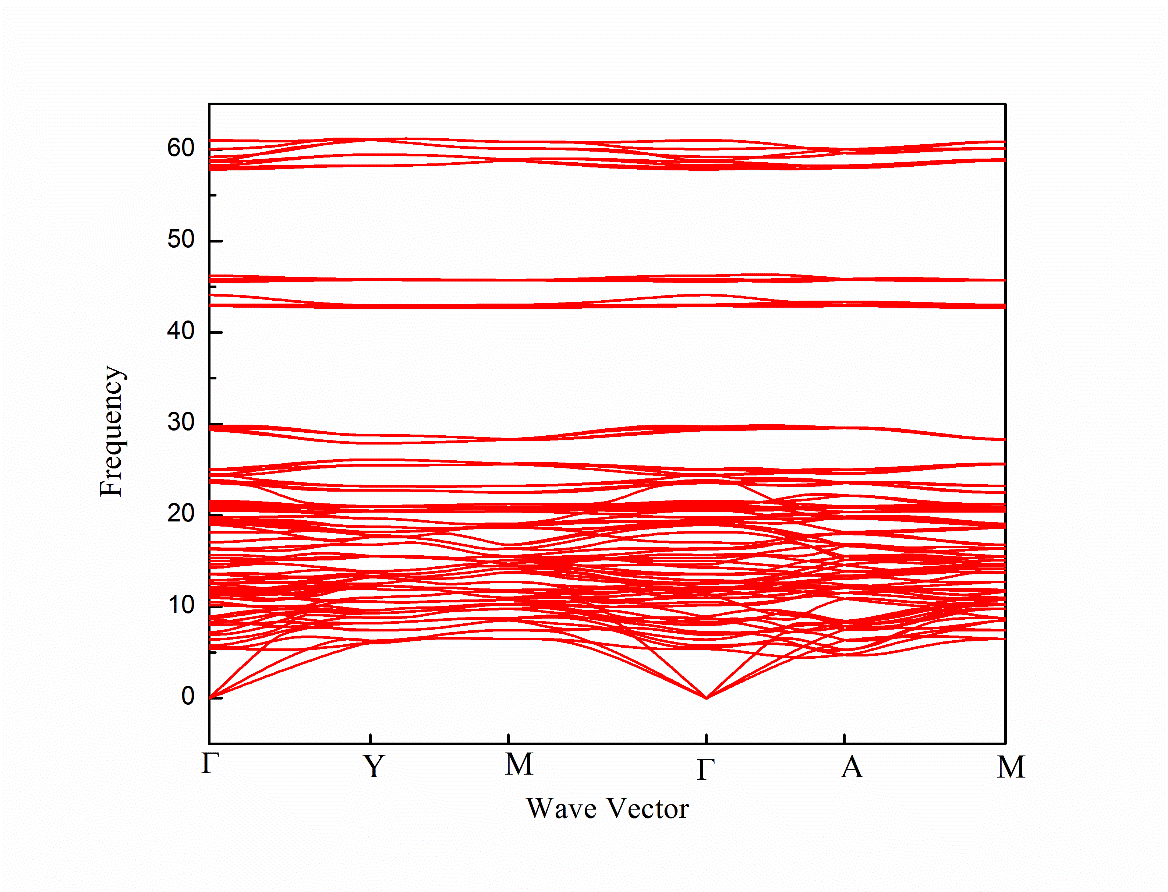


Figure S4 Phonon dispersion curves of *C*2*/c* N_2_O_5_


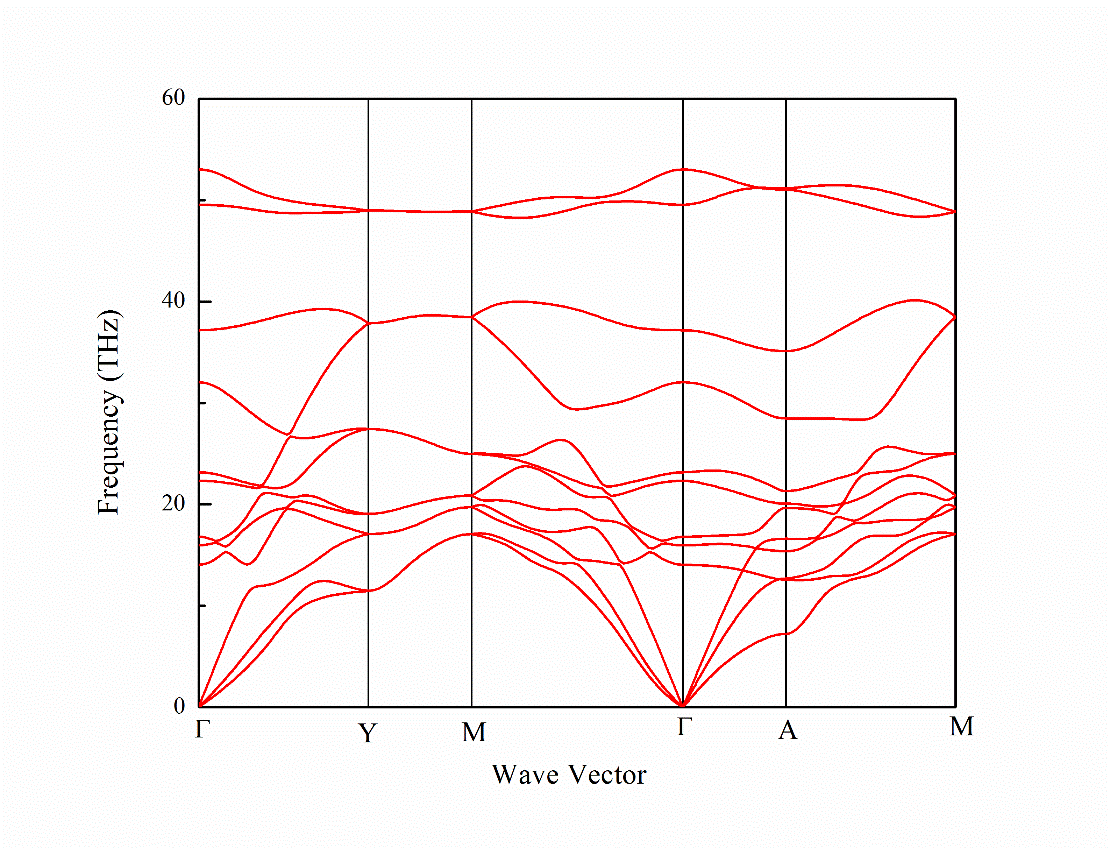


Figure S5 Phonon dispersion curves of *P*2_1_*/m* NO at 198 GPa. Γ(0,0,0), Y(0.5,0.5,0), M(0.5 0.5 0.5) and A(0,0.5,0.5)


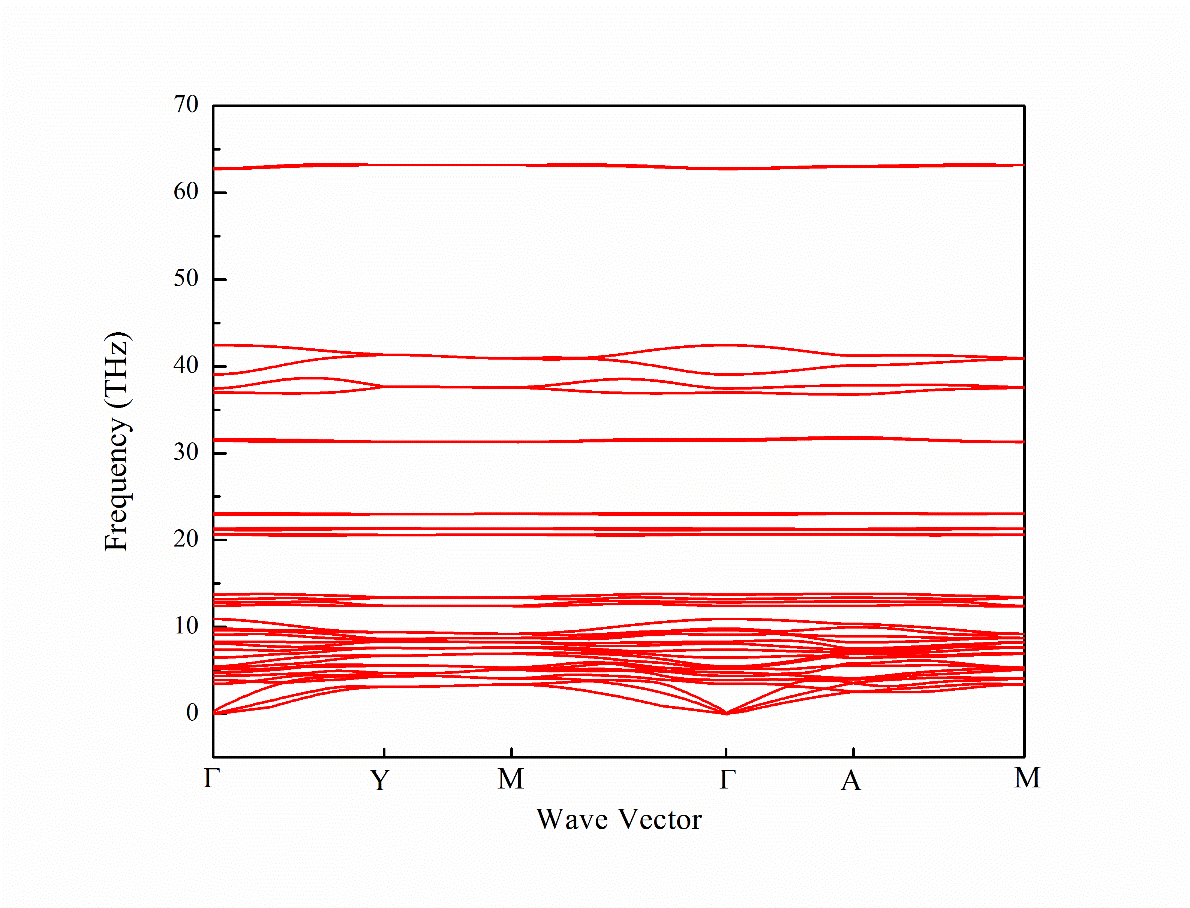


Figure S6 Phonon dispersion curves of *P*2_1_ NO^+^NO_3_^-^.


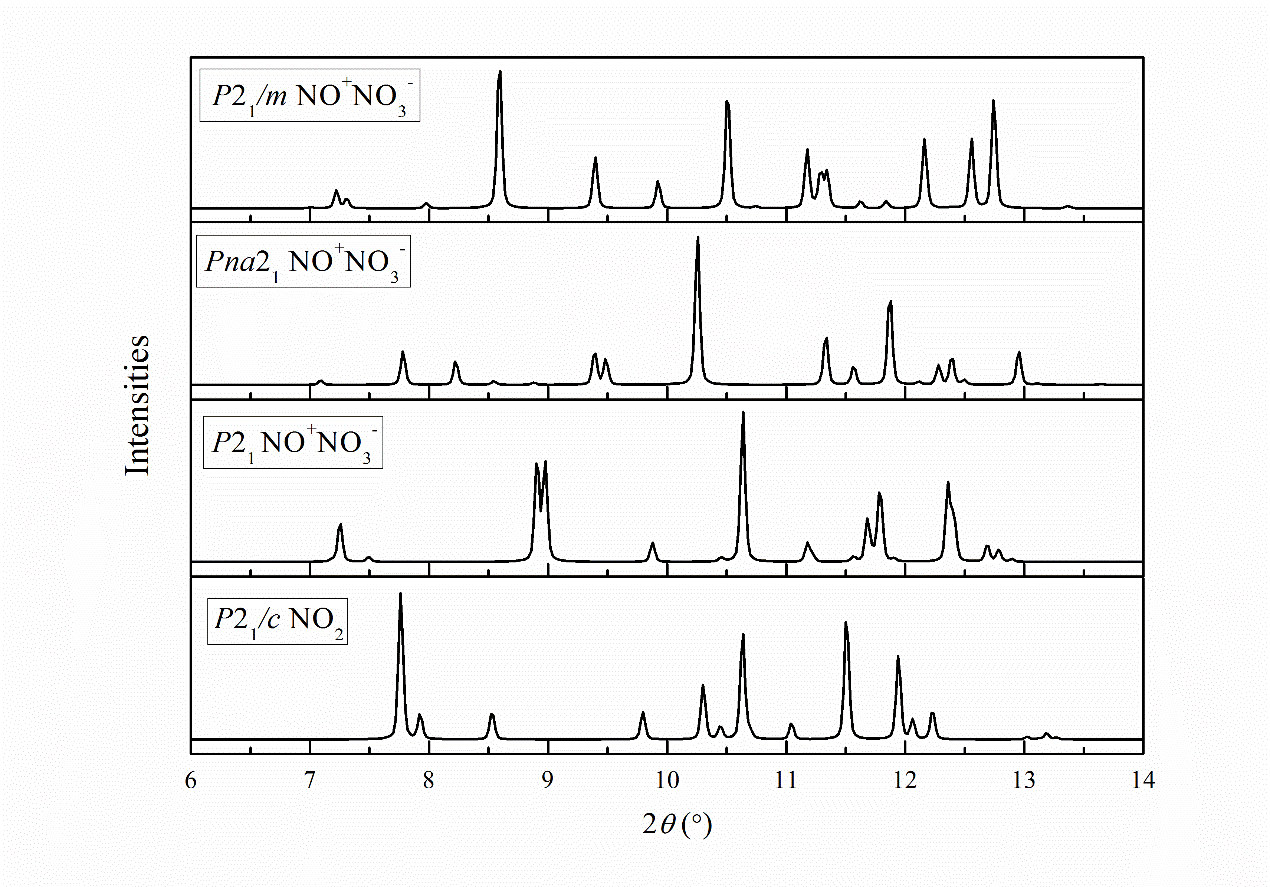


(a)


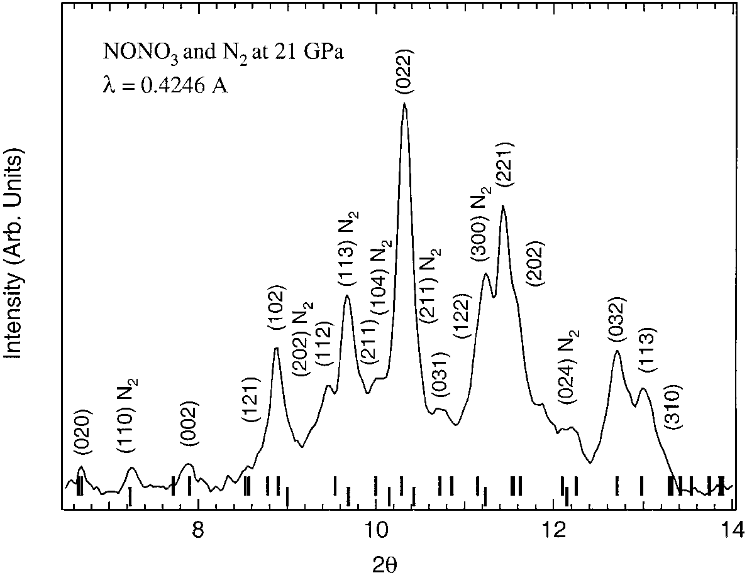


(b)

Figure S7 (a) X-Ray Diffraction data of *P*2_1_*/m*^1^, *Pna*2_1_^2^, *P*2_1_ NO^+^NO_3_^-^ and *P*2_1_*/c* NO_2_ simulated at 20 GPa (*λ*=0.4246 *Å*) (b) XRD data of NO^+^NO_3_^-^ in experiments reported by Somayazulu et al.^3^

1 Meng, Y. *et al.* Hard x-ray radiation induced dissociation of N_2_ and O_2_ molecules and the formation of ionic nitrogen oxide phases under pressure. *Physical Review B* **74,** doi:10.1103/PhysRevB.74.214107 (2006).

2 Xiao, H., An, Q., Goddard, W. A., 3rd, Liu, W. G. & Zybin, S. V. Formation of the -N(NO)N(NO)- polymer at high pressure and stabilization at ambient conditions. *Proc Natl Acad Sci USA* **110**, 5321-5325(2013).

3 Somayazulu, M. *et al.* Novel broken symmetry phase from N_2_O at high pressures and high temperatures. *Physical Review Letters* **87**, 135504 (2001). Doi 10.1103/Physrevlett.87.135504
